# Menopause-Associated Depression: Impact of Oxidative Stress and Neuroinflammation on the Central Nervous System—A Review

**DOI:** 10.3390/biomedicines12010184

**Published:** 2024-01-15

**Authors:** Gengfan Liang, Audrey Siew Foong Kow, Rohana Yusof, Chau Ling Tham, Yu-Cheng Ho, Ming Tatt Lee

**Affiliations:** 1 Faculty of Pharmaceutical Sciences, UCSI University, Kuala Lumpur 56000, Malaysia; 2 Faculty of Applied Sciences, UCSI University, Kuala Lumpur 56000, Malaysia; 3 Department of Biomedical Sciences, Faculty of Medicine and Health Sciences, Universiti Putra Malaysia, Serdang 43400, Selangor, Malaysia; 4 Natural Medicines and Products Research Laboratory (NaturMeds), Institute of Bioscience, Universiti Putra Malaysia, Serdang 43400, Selangor, Malaysia; 5 School of Medicine, College of Medicine, I-Shou University, Kaohsiung City 82445, Taiwan; 6 Centre of Research for Mental Health and Well-Being, UCSI University, Kuala Lumpur 56000, Malaysia

**Keywords:** estrogen deprivation, psychological wellbeing, pro-inflammatory cytokines

## Abstract

Perimenopausal depression, occurring shortly before or after menopause, is characterized by symptoms such as emotional depression, anxiety, and stress, often accompanied by endocrine dysfunction, particularly hypogonadism and senescence. Current treatments for perimenopausal depression primarily provide symptomatic relief but often come with undesirable side effects. The development of agents targeting the specific pathologies of perimenopausal depression has been relatively slow. The erratic fluctuations in estrogen and progesterone levels during the perimenopausal stage expose women to the risk of developing perimenopausal-associated depression. These hormonal changes trigger the production of proinflammatory mediators and induce oxidative stress, leading to progressive neuronal damage. This review serves as a comprehensive overview of the underlying mechanisms contributing to perimenopausal depression. It aims to shed light on the complex relationship between perimenopausal hormones, neurotransmitters, brain-derived neurotrophic factors, chronic inflammation, oxidative stress, and perimenopausal depression. By summarizing the intricate interplay between hormonal fluctuations, neurotransmitter activity, brain-derived neurotrophic factors, chronic inflammation, oxidative stress, and perimenopausal depression, this review aims to stimulate further research in this field. The hope is that an increased understanding of these mechanisms will pave the way for the development of more effective therapeutic targets, ultimately reducing the risk of depression during the menopausal stage for the betterment of psychological wellbeing.

## 1. Introduction

Depression is characterized by a range of mood, cognitive, and behavioral symptoms, which may lead to distress and functional impairment. These symptoms include persistent feelings of sadness and hopelessness, decreased interest or pleasure, changes in appetite, sleep disturbances, fatigue, feelings of worthlessness or excessive guilt, difficulty concentrating, psychomotor agitation or slowing, and recurrent thoughts of death [1]. In recent years, advancements in medical technology have led to a growing belief among scholars that depression is not merely a psychological disorder but also a neurological disease. Several hypotheses have been proposed regarding the underlying mechanisms of depression, including the monoamine hypothesis, neuroinflammation and oxidative damage, HPA axis abnormalities, and the neurogenesis hypothesis [2,3,4]. Major Depressive Disorder (MDD) is twice as prevalent in women of reproductive age compared to men [5]. Epidemiological studies have shown that approximately 20% of women may experience an episode of MDD at some point in their lives [6]. Furthermore, the risk of depression appears to increase during dynamic hormonal flux periods such as the perimenopause stage [7], with rates of MDD and clinical elevations in depressive symptoms doubling to tripling when compared to premenopausal and late postmenopausal rates [8]. Perimenopausal depression is a type of depression that occurs during the perimenopausal period, which is characterized by the simultaneous occurrence of multiple menopausal symptoms that overlap with emotional disorders, like depression and no interest, sleep disorders, tiredness, low energy, no self-confidence, and thoughts of death [9]. Based on the epidemiological studies, due to the differences in culture, dietary habits, and economic conditions, there were large differences in rates of psychologic symptoms in perimenopausal women across different countries. For instance, although perimenopausal women experience similar menopausal symptoms such as hot flushes, sweating, palpitations, dizziness, anxiety, irritability, headache, depression, and insomnia in all the countries, the proportion of women in East and Southeast Asian countries experiencing hot flushes and night sweating were generally lower than those observed in western countries [10]. On the other hand, the most common mental problems for Asian perimenopausal women were insomnia and irritability (51%). Additionally, stiff joints were a common symptom perceived negatively in Asian perimenopausal women but unusual in studies of women of other nationalities [11]. Furthermore, the incidence rate of depressive disorder among perimenopausal women in the same area is also affected by their educational levels and economic conditions; women with higher education levels living in urban cities were more susceptible to the symptoms of menopause than primary educated women in rural areas [12]. These epidemiological data underscore the significant burden of mental illnesses and suicidality that women carry [13]. Considering the exponential growth in the elderly population worldwide, especially among women at the menopausal stage, the healthcare and financial burden of perimenopausal depression is substantial [14,15,16]. Furthermore, menopausal depressive symptoms are highest during the early perimenopause period [17], and thus perimenopause presents a better opportunity for effective treatment of menopausal depression than the late menopausal period.

Perimenopausal depression is currently managed with conventional antidepressant medications, including several primary classes: tricyclic antidepressants (e.g., Clomipramine, amitriptyline, doxepin), monoamine oxidase inhibitors (e.g., Phenylethylhydrazine, bromofaromine, toloxadone, isozohydrazine), and selective serotonin reuptake inhibitors (e.g., fluoxetine, citalopram, escitalopram, sertraline) [18,19,20]. Additionally, gonadal hormones, such as estrogen treatment, are employed in some cases [17]. These medications aim to slow down the progression of depression and alleviate its symptoms. In recent years, novel pharmacotherapeutic agents such as serotonin/norepinephrine reuptake inhibitors (SNRIs), glutamatergic agents (such as ketamine and d-cycloserine), and selective estrogen receptor modulators (SERM) for treating perimenopausal depression have also gradually been used [21,22]. These new pharmacotherapeutics often have better efficacy or milder side effects. Overall, it is of paramount clinical significance and practical application value to conduct research into the pathological mechanisms underlying perimenopausal depression. Identifying potential therapeutic targets can lead to more effective interventions and improve the overall management of this condition.

The links between depression and cardiovascular disease are bidirectional. The incidence rate of depression in patients with cardiovascular disease is two to three times that of the general population [23], and depression is also a risk factor for the incidence rate and mortality of coronary heart disease or cardiovascular disease in patients with coronary heart disease [24]. Cardiovascular diseases are associated with an incidence risk of depression as cardiovascular agents. The main cardiovascular drugs currently used in clinical practice include lipid-lowering drugs, antiplatelet drugs, ACEIs, ARBs, β-blockers, angiotensin receptor-neprilysin inhibitor (ARNI), CCBs, diuretics, and nitrate drugs. Some cardiovascular drugs have antidepressant effects, while others may exacerbate depressive symptoms [25].

Lipid-lowering drugs are mainly used to treat high cholesterol and cardiovascular diseases, with statin-based lipid-lowering drugs being the most widely used. Research has found that statins are able to provide protection against depression by improving blood-flow, reducing coagulation, modulating the immune system, and reducing oxidative damage [26]. In animal experiments, atorvastatin alleviated lipopolysaccharide-induced depressive status in mice by reducing TNF-α levels and oxidative stress while increasing BDNF levels in the hippocampus and prefrontal cortex [27]. In clinical trials, it has also been found that statin drugs may downregulate IL-1β and NF-κB as anti-inflammatory drugs for the treatment of depression in patients with coronary heart disease [28].

β-receptor blockers are a class of drugs widely used to treat hypertension, angina, and arrhythmia. Compared to statins, the links between β-blockers and depression have been mixed. In a clinical trial, it was found that metoprolol treatment can exacerbate depression and high fatigue symptoms in patients with chronic heart failure and clinical psychiatric disorders [29]. But in a multicenter study, the prescription of β-receptor blockers is not associated with increased depressive symptoms or depressive disorders in the first year after myocardial infarction [30]. Some scholars believe that the differences in these results may be related to the quantity of β-blocker crossing the blood–brain barrier, since hydrophilic β-receptor blockers such as atenolol have a lower incidence of side effects, while lipophilic β-receptor blockers such as indole alcohol and propranolol typically have a higher incidence of side effects [31].

Angiotensin-converting enzyme inhibitors (ACEI)/angiotensin receptor blockers (ARBs) play a crucial role in regulating the renin–angiotensin–aldosterone system (RAAS) by regulating the synthesis and release of angiotensin to regulate blood pressure. In addition to regulating blood pressure, the renin-angiotensin system is also an important regulator of the nerve system [32]. Clinical trials showed that after taking captopril and propranolol for 6 months, white men with mild hypertension showed improvements in their cognitive function and social participation [33]. Similarly, a clinical trial in Europe also found that captopril can reduce the trend of depression symptoms in patients [34]. Angiotensin receptor-neprilysin inhibitors (ARNIs), such as sacubitril and valsartan, are similar to ACEIs/ARBs, which significantly improve the anxiety and depression index of heart failure patients. ARNI may reduce systemic inflammation and inhibit the RAAS and neprilysin pathway, which can potentially explain the alleviation of depressive symptoms [35].

## 2. Hormonal Changes during Perimenopause

Estrogen plays an important role in perimenopausal depression by participating in the regulation of neural circuits such as the serotonin, noradrenergic, and dopaminergic systems [36]. The neuroprotective effect of estrogen has been demonstrated in schizophrenia and neurodegenerative diseases like Alzheimer’s disease and Parkinson’s disease [17,37]. Clinical trials have shown that the blind cessation of hormone therapy in women with perimenopausal depression who have previously responded to hormone therapy can lead to the recurrence of depressive symptoms [38]. Another study found that supplementing estrogen to perimenopausal depression patients can improve their physical and depressive symptoms [39]. The above results indicate that the fluctuation and decrease in estrogen levels in perimenopausal women, and the subsequent weakening of estrogen neuroprotective effects may have negative psychological effects on some women.

During the early stages of menopause, one of the primary features of reproductive aging is the shortening of the menstrual cycle due to a reduction in the follicular phase. This phase involves processes such as follicle recruitment, growth, selection, and estrogen synthesis [40]. Follicle-stimulating hormone (FSH), a gonadotropin, plays a pivotal role in this process by accelerating follicular development and stimulating granulosa cells [40]. In the early stages of menopause, women exhibit higher FSH concentrations in the early follicular phase but possess fewer small follicles compared to younger women. This increase in FSH compensates for the decreased follicle count in older women and helps maintain stable estrogen levels through enhanced aromatase activity [41]. As menopause progresses, ovarian sensitivity to estrogen diminishes, and ovarian follicular depletion occurs, leading to a relatively hypoestrogenic environment. Despite menstrual cycle irregularities, some women may retain the ability to ovulate, resulting in increased progesterone production and potential fertility. This period is characterized endocrinologically by persistently high gonadotropin levels, sustained irregular menstruation with periods of amenorrhea, and hypoestrogenism [42]. In the postmenopausal stage, with the depletion of ovarian follicles, FSH levels continue to rise, while estradiol levels steadily decrease until they stabilize [43].

The decline in ovarian function and prolonged estrogen deprivation during the menopausal transition are known to affect psychological and cognitive wellbeing [7]. Research has shown that female patients in the menopausal transition and early postmenopausal years are more likely to experience depressive moods than premenopausal female patients [8,44]. Therefore, the menopausal transition and perimenopausal stage are critical periods during which female patients may be susceptible to anxiety and depressive disorders. The potential neuroendocrine mechanisms underlying how the complex hormonal changes during menopause may contribute to depressive symptoms remain unclear. Multiple hypothetical mechanisms are under investigation, including monoaminergic deficiency, reduction in neurotrophic factors, hypothalamic-pituitary-adrenal (HPA) axis dysfunction [45], oxidative stress [46,47], and low-grade chronic inflammation [48,49] during perimenopause.

## 3. Monoamine Hypothesis

The monoamine hypothesis of major depression was formulated almost fifty years ago. The monoamine hypothesis, rooted in the discovery of the antidepressant effects of monoamine oxidase inhibitors (MAOIs), has played a significant role in our understanding of depression. The monoamine hypothesis postulated that depression results from low levels of serotonin, norepinephrine, and/or dopamine in the central nervous system (CNS) [50]. MAOIs and tricyclic antidepressants (TCAs) were found to alleviate depressive symptoms by increasing the concentration of these neurotransmitters [51]. In addition, lower plasma tryptophan levels (the precursor of serotonin) and reduced serotonin levels in cerebrospinal fluid have been observed in patients with depression; therefore, serotonin is also included in this hypothesis and formed the classical monoaminergic hypothesis, which has also resulted in the design of the selective serotonin reuptake inhibitor (SSRI) class of antidepressants [52]. Although there seems to be a consensus among people that increasing monoamine levels can alleviate depressive symptoms, a more comprehensive understanding of the monoamine hypothesis and new drug targets targeting the central monoamine system is still under investigation [50,51,52]. Estradiol plays a role in modulating serotonin synthesis, increasing 5-hydroxytryptamine receptor 2A (5-HT_2A_) expression, and diminishing serotonin catabolism [53]. It accomplishes this through the regulation of tryptophan hydroxylase type 2 gene expression and the reduction in monoamine oxidase-A (MAO-A) levels and enzyme activity [54,55]. Brain imaging studies have demonstrated higher MAO-A levels in women at the perimenopausal age than at the premenopausal age, suggesting a potential connection with changes in female gonadal hormones [56], which is in line with an earlier observation that associated gonadal and stress hormones with serotonin deficiency in women with depression [57].

## 4. Perimenopausal Depression and BDNF Deficiency

Brain-derived neurotrophic factor (BDNF), a 119-amino acid secretory polypeptide, is widely distributed in various regions of the central nervous system, including the cortex, hippocampus, visual cortex, striatum, substantia nigra, retrorubral region, and ventral tegmental area [58]. BDNF plays a critical role in memory formation and hippocampal plasticity, which is a fundamental neural correlation of learning and memory. The production of BDNF involves a highly regulated process. The *BDNF* gene generates numerous *BDNF* mRNA transcripts, which ultimately give rise to the mature isoform of BDNF after a sequence of events. This process involves the translation of all transcripts into the same pre-pro-BDNF protein in the endoplasmic reticulum, subsequent transport to the Golgi apparatus, cleavage to form pro-BDNF, and final cleavage to produce mature BDNF [59]. It is important to note that pro-BDNF and mature BDNF can interact with different receptors, activating distinct intracellular pathways and exerting varying effects [60].

Preclinical evidence has shown that idecreased BDNF levels in the hippocampus and prefrontal cortex (PFC) is associated with bilateral ovariectomy, leading to effective depression due to estrogen deficiency in mice [61,62]. Further research has indicated that estrogen may increase BDNF expression and release through estrogen receptor β, mediating its regulatory role [63]. Mature isoforms of BDNF can bind to the tyrosine kinase receptor B (TrkB), promoting cell survival and enhancing hippocampal long-term potentiation. After BDNF binds to TrkB on the cell membrane, several signaling pathways are activated, including PI3K/Akt, MEK/ERK, and LKB1/AMPK. These pathways subsequently affect the mammalian target of rapamycin complex-1 (mTORC1), a crucial growth regulator [64,65]. The activation of mTORC1 has several outcomes, including increased synaptic formation, enhanced neural plasticity, and rapid antidepressant effects [66,67]. Additionally, the mTOR signaling pathway regulates autophagy and promotes nerve regeneration [68], which regulates depression through the bidirectional control of autophagy and autophagic flux [69]. The AMPA receptor-BDNF-mTOR signal pathway is suggested to be essential for enhancing synaptic function in the medial prefrontal cortex and contributes to the rapid antidepressant effect of ketamine [70].

Previous studies have highlighted the involvement of neurotrophins like BDNF in hypothalamic-pituitary-adrenal (HPA) axis neuroendocrine regulation [71]. Substances that upregulate BDNF were shown to mitigate the effects of chronic stress-induced depression by ameliorating the HPA axis and the hippocampal glucocorticoid receptor (GR) dysfunctions [72] as well as the 5-HT system [73]. This complex interplay among neuropsychological behaviors, BDNF expression, and the HPA axis was also implicated in perimenopause-associated depression, in relation to inflammatory factors [74]. Clinical studies have demonstrated that C-reactive protein and TNF-α are positively correlated with self-rating depression scale scores, while BDNF levels are negatively correlated with these scores in women with perimenopausal syndrome [74]. Furthermore, the level of serum estradiol is significantly positively correlated with BDNF levels, indicating that a progressive reduction in estradiol in peripheral blood is associated with decreased BDNF serum quantity in perimenopausal women [75].

## 5. Perimenopausal Depression and HPA Axis Dysregulation

**Figure 1:**
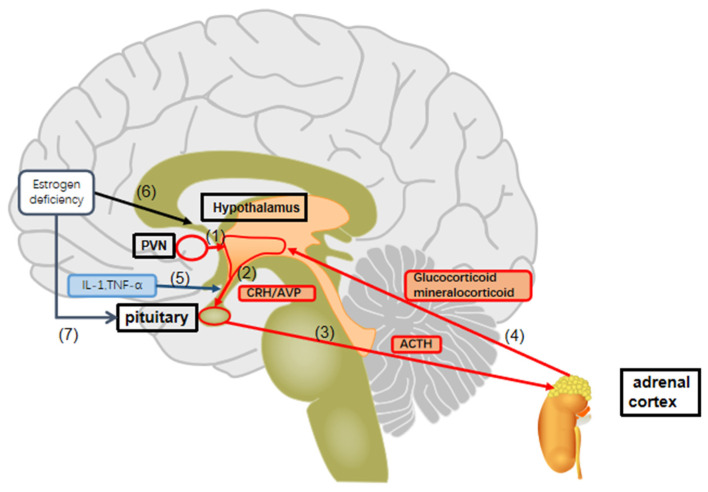
Estrogen deficiency and HPA axis dysregulation. (1) Neurons in the hypothalamus release the hypothalamic neuropeptides corticotrophin-releasing hormone (CRH) and arginine vasopressin (AVP). (2) CRH and AVP promote a posttranslational cleavage of anterior pituitary pro-opiomelanocortin mRNA (POMC), resulting in the synthesis and secretion of adrenocorticotrophin (ACTH). (3) ACTH stimulates the release of glucocorticoids and mineralocorticoids derived from the adrenal glands. (4) The hippocampus, as an important site of glucocorticoid receptor (GR) and mineralocorticoid (MR) receptors mediated negative feedback regulation, plays a vital role in the end stage of this stress feedback process. (5) TNF-α and IL-1β trigger the imbalance of the HPA axis by inhibiting the transcriptions of c-fos and AVP. (6) Estrogen is able to regulate the hypothalamic response to stress by regulating CRH, oxytocin, and AVP gene expression in hypothalamic neurons and the excitatory inputs to the PVN. Perimenopausal estrogen deficiency leads to weakened regulation of the HPA axis response to external stress. (7) Estrogen can block the actions of CRH on anterior pituitary corticotrophs by increasing the expression of CRH-BP and restricting the amounts of bioactive CRH. Perimenopausal estrogen deficiency increases the sensitivity of the pituitary gland to CRH, thereby resulting in the overactivation of the HPA axis.

The hyperactivity of the HPA axis is closely associated with the pathophysiology of anxiety, depression, and cognitive functioning [76,77,78]. This process encompasses a series of forward and feedback inhibition loops involving the brain, pituitary gland, and adrenal glands. When individuals perceive stress or social threats, it triggers the activation of the HPA axis. This activation involves the amygdala and amplification of stress responses within the paraventricular nucleus (PVN), potentially leading to anxious states. Neurons in the PVN and hippocampus subsequently release hypothalamic neuropeptides, including corticotrophin-releasing hormone (CRH) and arginine vasopressin (AVP). These neuropeptides facilitate the posttranslational cleavage of pro-opiomelanocortin mRNA (POMC) in the anterior pituitary, resulting in the synthesis and secretion of adrenocorticotrophin (ACTH). ACTH is released into the circulatory system, stimulating the release of glucocorticoids and mineralocorticoids from the adrenal glands [79,80]. These hormones circulate in both peripheral blood and cerebrospinal fluid and bind to intracellular nuclear steroid receptors (Figure 1).

The hippocampus, one of the key regions with glucocorticoid receptor (GR) and mineralocorticoid receptor (MR) expression, plays a crucial role in negative feedback regulation of the stress response [81,82]. MR receptors initiate the stress response, while GR receptors in the hippocampus, PVN, and anterior pituitary gland terminate the stress response. Dysfunctional cortisol negative feedback, which can lead to hypercortisolism in both acute and chronic major depression, is believed to be responsible for HPA axis abnormalities in MDD (Figure 1) [83].

A close relationship exists between the activities of the HPA axis and the hypothalamic-pituitary-gonad (HPG) axis, and they can interact in estrogen-mediated affective disorders. The central nervous system can regulate the synthesis and secretion of estrogen through the HPG axis. Conversely, estrogen plays a significant role in the onset and course of female MDD. For example, it has been reported that contraceptive use can impact the degree of positive effect change in women’s daily experiences [84]. Clinical studies have also indicated that estrogen facilitates the action of selective serotonin reuptake inhibitors (SSRIs), increasing the sensitivity of perimenopausal depression patients to SSRIs and improving treatment outcomes [85]. Additional research using estrogen antagonists demonstrated an increase in ACTH and corticosterone responses to restraint stress in female mice. Conversely, in ovariectomized models simulating menopause, low-dose estradiol decreased the stress-induced ACTH response [86]. These findings support the notion that estrogen can regulate and modulate the HPA axis by inhibiting its overactivation (Figure 1).

In a study involving 30 perimenopausal women, an increase in estradiol levels was found to be associated with an increase in cortisol levels. Both estradiol and cortisol levels were also correlated with negative mood in women who had experienced depression in the past and those currently experiencing depressive symptoms [87]. In other words, the fluctuation in estradiol levels may play a role in perimenopausal depression through HPA axis dysregulation.

## 6. Effect of Neuroinflammation in Perimenopausal Depression

Two prominent features of perimenopause, bioenergetic deficits and chronic inflammation, have been identified as unifying factors that causally connect genetic risk factors for MDD and various neurodegenerative diseases [88,89,90]. Among the serum biomarkers associated with perimenopause is a significant upregulation of pro-inflammatory cytokines secreted by CD4 T cells, such as interleukin (IL)-8 and tumor necrosis factor-alpha (TNF-α) [91]. Research has shown that estradiol levels in peripheral serum have an inverse relationship with serum IL-8, TNF-α, as well as microglial and astrocytic reactivity in perimenopausal women [92]. Additionally, the decline in estrogen levels can lead to immune cell activation, and the resulting pro-inflammatory cytokine milieu, including IL-1, IL-6, and TNF-α [93,94]. Once released into the bloodstream, these peripheral cytokines can directly or indirectly reach the brain through various pathways:

Leaky Circumventricular Regions: Cytokines may pass through leaky circumventricular regions, such as the area postrema or the subfornical organ, in the blood–brain barrier.

Active Transport: Some cytokines might be transported through saturable transport molecules.

Endothelial Activation: Cytokines can activate endothelial cells and other cell types, like perivascular macrophages, lining the cerebral vasculature, which then produce cytokines and other inflammatory mediators.

Nerve Fiber Signaling: Cytokines can bind to cytokine receptors associated with peripheral afferent nerve fibers, such as the vagus nerve, which can then relay cytokine signals to the relevant brain regions [95].

**Figure 2:**
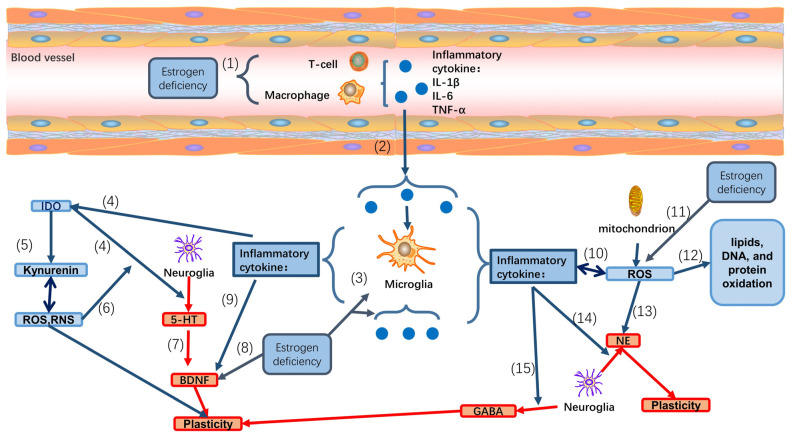
Neuroinflammatory pathway following estrogen deficiency. (1) Estrogen can inhibit the secretion of inflammatory factors by T cells and macrophages, and the decrease in estrogen levels leads to chronic inflammation during perimenopause, resulting in an increase in inflammatory mediators in peripheral blood. (2) These peripheral cytokines will directly or indirectly reach the brain via different specific or non-specific pathways. (3) In premenopause, β-estradiol inhibits microglia-mediated astrocytic activation to alleviate neuroinflammation and the secretion of inflammatory mediators by microglia. On the other hand, in perimenopause, estrogen deficiency allows inflammatory cytokines to activate microglia in the brain, which then produce other inflammatory mediators. (4) IL-β, IL-6, and TNF-α reduce the synthesis of 5-HT by stimulating indoleamine 2,3-dioxygenase (IDO). (5) IDO can convert tryptophan into kynurenine, and the activation of the kynurenine pathway results in the production of ROS and RNS. On the other hand, superoxide anion radicals can cause superinduction of the kynurenine pathway. (6) ROS and RNS reduce the synthesis of 5-HT by stimulating IDO and the kynurenine pathway. (7) Neuroglia secrete serotonin, which then promotes BDNF secretion. (8) Similarly, estrogen can promote the synthesis and secretion of BDNF; therefore, after menopause, insufficient estrogen can lead to a decrease in BDNF synthesis. (9) IL-β, IL-6, and TNF-α can inhibit the neuroprotective effect of BDNF. (10) IL-β, IL-6, TNF-α can promote the production of ROS in mitochondria. Furthermore, the ROS can also promote the synthesis and release of inflammatory factors. (11) Estrogen can reduce ROS production by promoting the synthesis of antioxidant enzymes, and insufficient estrogen during perimenopause leads to an increase in ROS. (12) ROS can lead to lipid, DNA, and protein oxidation and damage in the brain. (13) ROS reduces the production of monoaminergic neurotransmitters and other aminergic compounds. (14) IL-β decreases the rate-limiting enzyme in noradrenaline. (15) IL-β, IL-6, and TNF-α can suppress GABAergic inhibitory transmission.

Microglial Involvement: Microglia can produce CC-chemokine ligand 2 (CCL2) and CXC-chemokine ligand 1 (CXCL1), which can facilitate the entry of immune cells into the cerebrospinal fluid (CSF) (Figure 2) [96].

Interleukin-1beta (IL-1β) is a member of the interleukin-1 family, which comprises a group of circulating cytokines known for their involvement in inflammation and various diseases. There are 11 members in the IL-1 family expressed by several immune cells, including microglial cells, dendritic cells, mononuclear cells, neutrophils, T cells, and B cells [97]. These immune-modulating cytokines can induce inflammatory reactions in various cell types and tissues. Some interleukins are associated with pro-inflammatory effects (e.g., IL-1α, IL-1β, IL-18, IL-36α, IL-36β, and IL-36γ), while others have anti-inflammatory properties (e.g., IL-1Ra, IL-33, IL-36 Ra, IL-37, and IL-38). The biological functions of these interleukins have been linked to many disease processes, including MDD [98,99].

The hippocampus, a crucial part of the limbic system located between the thalamus and the medial temporal lobe, plays a vital role in spatial and contextual memory. In the hippocampus, IL-1β, its receptor (IL-1R), and the natural interleukin-1 receptor antagonist (IL-1RA) are expressed at relatively high levels, indicating their potential to regulate hippocampal memory functions. This supports the hypothesis that excessive or dysregulated IL-1 signaling may contribute to deficits in hippocampus-dependent memory processes [100,101]. Pro-inflammatory cytokines, such as TNF-α and IL-1β, can influence responses to glutamate, a neurotransmitter that can be converted into gamma-aminobutyric acid (GABA) by the enzyme glutamic acid decarboxylase (GAD) [102]. This influence by pro-inflammatory cytokines occurs through complex effects on N-methyl-D-aspartate (NMDA) and α-amino-3-hydroxy-5-methyl-4-isoxazolepropionate (AMPA) receptor-associated function [103]. Chronic elevations in IL-1β, induced by intraventricular infusions of lipopolysaccharide (LPS) for more than 28 days, have been found to reduce NMDAR-dependent and NMDAR-independent forms of hippocampal long-term potentiation (LTP). LTP is a critical substrate for hippocampus-dependent memory and is evoked by high-frequency and theta burst stimulation [104]. In the experimental autoimmune encephalomyelitis (EAE) mouse model, IL-1β secreted by activated microglia was reported to suppress GABAergic inhibitory transmission, potentially leading to neuroplastic injury [105]. Additionally, in animal models of aging, IL-6 has been shown to regulate the age-related loss of key parvalbumin-expressing GABAergic interneurons by increasing neuronal NADPH-oxidase-derived superoxide production. This effect preserves cognitive performance in aging mice (Figure 2) [106].

Several cytokines, including IL-1, interferon-gamma (IFN-γ), and TNF-α, have profound effects on the central nervous system. Studies have shown that intranigral injection of LPS increased the concentrations of serotonin catabolite and 5-hydroxyindoleacetic acid (5-HIAA) in various brain areas, while significantly decreasing the levels of dopamine and its metabolites, such as dihydroxyphenylacetic acid [107]. Furthermore, research has revealed that cytokines not only acutely stimulate serotonin neurotransmission but also reduce the synthesis of serotonin by activating indoleamine 2,3-dioxygenase (IDO), an enzyme that converts the precursor of serotonin into kynurenine. Overexpression of IDO leads to the depletion of plasma concentrations of serotonin precursors, subsequently reducing serotonin synthesis in the central nervous system [108]. In addition to the serotonin system, the influence of cytokines on the noradrenergic system has also been documented. IL-1β has been reported to decrease the activity of tyrosine hydroxylase, the rate-limiting enzyme in noradrenaline biosynthesis. Increases in noradrenaline levels correlate with increased tyrosine hydroxylase activity in the brain and mRNA levels in the nucleus tractus solitarius region, which suggests a potential contribution to the reduction in noradrenaline levels in the hypothalamus (Figure 2) [109].

Studies have shown that inflammatory cytokines such as IL-1β, IL-6, and TNF-α can impair neuronal cells by inhibiting the neuroprotective effects of BDNF [91]. In in vitro experiments, while IL-1β did not significantly alter the transcription and expression levels of the TrkB receptor, it attenuated the activation of the TrkB receptor and its downstream signaling pathways, including phospholipase Cγ1 (PLCγ1) and the p40/42 mitogen-activated protein kinase (MAPK) signaling pathway. This IL-1β-induced suppression of the BDNF neuroprotection process could be mitigated by inhibitors of ceramide production [110]. Moreover, transient elevations in IL-1β within the physiological range can enhance hippocampus-dependent memory. However, levels of IL-1β higher than the physiological range result in the upregulation of p38 MAPK, potentially disrupting BDNF-dependent synaptic plasticity processes. Inhibiting p38 MAPK can block these neural plasticity-damaging effects of IL-1β (Figure 2) [111].

Cytokines play a pivotal role in mediating the interaction between immune cells and the neuroendocrine system, particularly through the HPA axis and its effects on immunosuppression. Studies have indicated that IL-1β-induced increases in dialysate noradrenaline partly correspond to the increases in plasma adrenocorticotropic hormone (ACTH) and corticosterone levels, demonstrating a relationship between noradrenaline, IL-1β, and HPA axis activation in animal models [112]. Furthermore, reports have shown that one endocrine effect directly linked to IL-1 is the stimulation of the hypothalamic corticotropin-releasing factor, resulting in increased secretion of ACTH and glucocorticoids. This mechanism may lead to glucocorticoid resistance, disrupting HPA axis function and contributing to mood disorders [112,113]. In animal models of anxiety and depression, female mice exhibiting depressive behaviors, elevated inflammatory cytokines (such as TNF-α and IL-1β), and HPA axis activation, but reduced transcription of c-fos and AVP indicate that inflammatory factors may disrupt the balance of the HPA axis by inhibiting the transcription of c-fos and AVP [114].

In addition to the inflammatory cytokines mentioned above, some other cytokines such as transforming growth factor-β (TGF-β) also play certain roles in the occurrence of perimenopausal depression. As a new member of the cytokine family, TGF-β debuted with the rise of vertebrates, and almost all human cell types respond to TGF-β. TGF-β regulates the expanding systems of epithelial and neural tissues, the immune system, and wound repair by regulating cellular proliferation, differentiation, survival, adhesion, and the cellular microenvironment [115]. The abnormalities of TGF-β and its downstream signaling pathways are closely related to the occurrence of many diseases such as tumors, cardiovascular diseases, osteoarthritis, and neurodegenerative diseases [115,116,117]. Clinical brain-imaging evidence suggested that hippocampal volume is decreased in patients with depressive disorder. Subsequent animal experiments also found atrophy and loss of CA3 pyramidal neurons in rats’ hippocampus. On the contrary, chronic antidepressant treatment significantly promotes neurogenesis the hippocampus [118]. These results illustrated that neurogenesis plays a vital role in depression and can impact behavioral output and the efficacy of antidepressant therapy, which was also the simplest form of the neurogenesis hypothesis [119].

TGF-β plays an important role in neurogenesis due to its ability to regulate the proliferation, differentiation, and maturation of nerve cells through its classical and non-classical signaling pathways. TGF-β1 deficiency in adult mice led to increased susceptibility of neurons to excitotoxic damage and a widespread increase in degenerating neurons accompanied by reduced expression of synaptophysin and laminin. Prominent microgliosis was also found in neonatal TGF-β1-deficient mice [120]. TGF-β affects the occurrence and development of perimenopausal depression through the following pathways: (1) TGF-β maintains the rate of cell division of neuronal precursors in the adult brain and hence the amount of neurogenesis through the classic TGF-β/Smad3 pathway [121]. (2) Neurons with a knockdown of TGF-β activated kinase 1 (TAK1) had significantly shorter axons than normal neurons, while TGF-β rescued axonal growth via the TGF-β/TAK1 signal pathway; (3) TGF-β was involved in neuronal differentiation and axon growth through the TGF-β/MAPK signal pathway; (4) TGF-β2 increased CREB phosphorylation in hippocampal cells, which mediated the long-term improvement of depressive behavior by TGF-β2 [116].

The regulation of female gonadotropins is closely related to TGF-β. Follicle-stimulating hormone (FSH) production in premenopausal women is under ovarian negative feedback by estradiol and the ovarian peptides inhibin A (InhA) and inhibin B (InhB), which are related heterodimeric members of the TGF-β superfamily. And the increase in the FSH at the beginning of menopause is a result of a decrease in gonadal InhB levels, which is the first sign of ovarian dysfunction and diminished ovarian follicle number. With the loss of gonadal function in late perimenopausal women, a significant decrease in estrogen is accompanied by a decrease in the levels of TGF-β superfamily members, ultimately leading to reduced neurogenesis, decreased neural plasticity, and the occurrence of depression [122].

## 7. Perimenopausal Depression and Reactive Oxygen Species (ROS)

Oxidative stress is characterized by an imbalance between the production and elimination of reactive oxygen species (ROS) or nitrogen (RNS) species. Excessive ROS play a significant role in the pathogenesis of MDD [2,123,124], while acting as modulators of neuronal excitability under physiological conditions [125]. An apparent increase in the ratio of oxidants over antioxidants may lead to irreversible changes in the organism, resulting in tissue and cellular damage, which is common in various pathological states [125,126]. ROS can lead to oxidation and damage of lipids, DNA, and proteins in the brain. Brain tissue is particularly sensitive to oxidative stress due to its high oxygen consumption and the presence of high levels of cellular lipids, which are rich in saturated fatty acids and susceptible to oxidation by ROS [127,128]. Furthermore, highly reactive species formed within ROS, such as oxide ion (O^2−^), can interact with nitric oxide (NO) to form peroxynitrite, which impairs enzymatic function, tyrosine residues, and reduces the production of monoaminergic neurotransmitters and other aminergic compounds (e.g., Tetrahydrobiopterin (BH_4_)), which are necessary for monoamine synthesis [129,130]. On the other hand, superoxide anion radicals (SAR) like O^2−^ can act as a co-substrate of IDO, leading to the superinduction of the neurotoxic kynurenine pathway in the presence of excessive oxidative stress, significantly reducing the survivability of neuronal cells [108,131]. ROS thus serves as a potent driving force behind the neuroprogressive changes that impact neurogenesis and contribute to the pathophysiological link between mood disorders and neurodegenerative processes.

**Figure 3:**
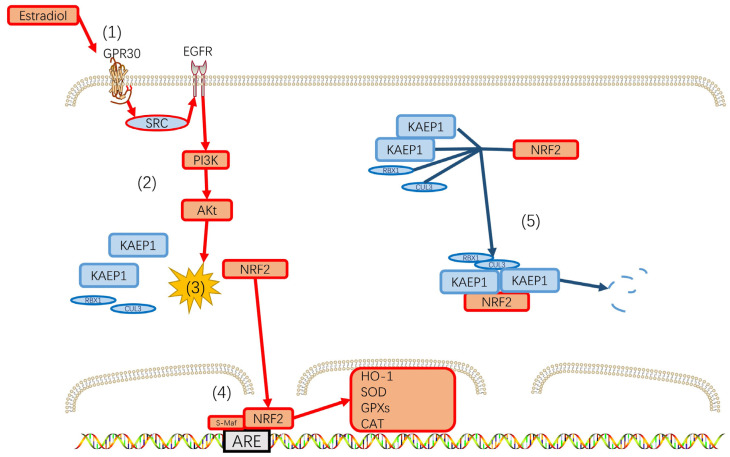
The antioxidant mechanism of estrodiol. (1) Estradiol binds to G-protein-coupled receptor 30 on the cell membrane. (2) GPR30 activation by estradiol elicited the SRC/EGFR/PI3K/Akt signaling pathway. (3) The activation of the GPR30/SRC/EGFR/PI3K/Akt signaling pathway leads to Nrf2 dissociation from Keap1. (4) Nrf2 translocates into the nucleus. Then the heterodimerization of NRF2 with small Maf proteins in AREs occurs and activates transcription of SOD, CAT, HO-1, GPxs, and so on. (5) Insufficient estrogen during perimenopause results in KEAP1-recruited CUL3, RBX, and NRF2 to degrade NRF2 through ubiquitination and ultimately leads to the decrease in antioxidant substances, while the increase in ROS leads to increased levels of oxidative stress and nerve damage in perimenopausal women.

Mitochondria are the primary intracellular organelles producing ROS, and the electron transport chain (ETC) complex I and ETC complex III generate O^2−^ during the electron transfer process [132,133]. Inflammatory cytokines, such as TNF-α, can stimulate the production of ROS in mitochondria by inhibiting the electron transport chain and altering membrane permeability [134,135]. Studies have shown that TNF-α increases ROS levels in microglia [136,137], and mitochondrial dysfunction in depression leads to elevated ROS [123]. Conversely, ROS can also promote the synthesis and release of inflammatory factors [138], which creates a vicious neuroinflammation–ROS cycle. To regulate the production of ROS, mitochondria produce several protective antioxidant molecules such as Heme oxygenase 1 (HO-1), glutathione peroxidases (Gpxs), catalase, and superoxide dismutase [139,140,141]. The transcription of these antioxidant molecules is significantly regulated by nuclear respiratory factor 2 (Nrf2). Under normal conditions, Kelch-like ECH-associated protein 1 (Keap1) recruits Nrf2 into the Cul3-containing E3 ubiquitin ligase complex, which ubiquitylates Nrf2 and leads to its degradation. In contrast, oxidative stress activates Nrf2 by inhibiting the E3 ubiquitin ligase activity, resulting in the dissociation between Nrf2 and Keap1. Nrf2 then translocates into the nucleus, and the heterodimerization of Nrf2 with small musculoaponeurotic fibrosarcoma (sMaf) proteins in antioxidant response elements (AREs), which are conserved gene sequences in the promoter regions of target genes, activates the transcription of these antioxidant enzymes [142,143]. Several studies have reported that Nrf2 activators, such as rice protein, melatonin, and other natural compounds, can attenuate depressive-like behaviors via the Keap1-Nrf2-ARE pathway [144,145]. In an animal model of glutamate-induced oxidative stress in rat brains, co-treatment of estradiol with glutamate can mitigate neuroinflammation, neurodegeneration, and synapse loss by reducing brain oxidative species through upregulation of the Nrf2/HO-1 antioxidant pathway and its downstream antioxidant molecules as well as the activation of pro-survival p-Erk1/2 MAP kinase pathways [146]. Interestingly, a recent study showed that activation of the Nrf2/HO-1 pathway significantly alleviated depressive-like behaviors in a menopause-mimicking model in mice (Figure 3) [147].

Cyclic GMP (cGMP) is synthesized by the soluble isoform of guanylyl cyclase (GC) in response to nitric oxide (NO) and carbon monoxide (CO) or natiuretic peptides (NPs), such as atrial, B-type, and C-type natiuretic peptides (ANPs, BNPs, and CNPs) [148]. The NO/cGMP signaling pathway participates in depression- and anxiety-related behaviors by regulating oxidative stress, neuroinflammation, and cortisol levels, among other mechanisms [149,150]. On the one hand, estrogen can increase cGMP by stimulating granulosa guanylate cyclase directly [151], and on the other hand, it can activate the NO/cGMP/PKG cascade reaction by increasing the synthesis of nitric oxide (NO), thereby achieving its various physiological functions [148]. After menopause, as estrogen levels decrease, cGMP levels in women’s bodies also decrease. A clinical trial found that postmenopausal women excreted significantly less urinary cGMP than premenopausal women [152].

cGMP can be hydrolyzed by enzymes of the PDE superfamily, which has been studied as a drug target for treating depression. PDE is divided into 11 different families (PDE1-11) and many drugs have been developed based on these PDE targets, such as the PDE5 inhibitor sildenafil [153]. Animal research also reported that Pde4d-KO mice exhibited decreased immobility in tail suspension and forced swim tests, which suggested that PDE4D plays a role in the pathophysiology and pharmacotherapy of depression [154]. Although there is still a lack of relevant clinical trials, this can serve as a new direction for the development of related antidepressant drugs in the future.

Through analyzing derivatives of reactive oxygen metabolites (d-ROMs), a marker of oxidative stress, and the biological antioxidant potential (BAP), an indicator of antioxidant protein levels, in women, it was found that oxidative stress was significantly higher in postmenopausal women than in premenopausal women [155]. Another clinical trial found that malonaldehyde (MDA), 4-hydroxynenal (4-HNE), and oxidized lipoproteins (ox LDL) were higher in postmenopausal than fertile women, while glutathione peroxidase levels were significantly higher in fertile women than in postmenopausal subjects, which indicated that the levels of oxidative stress in postmenopausal women increase while the synthesis of antioxidant enzymes decreases [156]. Furthermore, an increase in oxidative stress and the reduction in the mRNA expression of both SOD and GPxs were observed in women who underwent total hysterectomy with bilateral salpingo-oophorectomy, while estrogen replacement therapy is able to prevent and counteract such modifications by acting as a regulator of key antioxidant gene expression [157]. These results indicate that estrogen reduces oxidative stress levels in women by regulating the synthesis of antioxidant enzymes in the body.

## 8. Conclusions

The decline in estrogen and its protective effects, coupled with oxidative stress, make perimenopausal depression a significant risk for women in the menopausal stage. With evidence suggesting that women in the perimenopausal stage are at a considerable risk of developing depression, it is a critical issue that must be addressed to alleviate the burden on affected individuals and the healthcare system. In this manuscript, we have presented some advances in the pathological research of perimenopausal depression. We believe that these findings could provide valuable insights and potential therapeutic targets for the development of antidepressant drugs in the future, particularly for the population inflicted with menopause-associated depressive disorder.

## Data Availability

Not applicable. The authors employed generative AI tools for proofreading and language enhancement purposes.

## Abbreviation

4-HNE	4-hydroxynenal
5-HIAA	5-hydroxyindoleacetic acid
5-HT_2A_	5-hydroxytryptamine receptor 2A
ACEI	Angiotensin-converting enzyme inhibitors
ACTH	Adrenocorticotrophin
AMPA	α-amino-3-hydroxy-5-methyl-4-isoxazolepropionate
ANP	Atrial natiuretic peptide
ARBs	Angiotensin receptor blockers
AREs	antioxidant response elements
ARNIs	Angiotensin receptor-neprilysin inhibitor
AVP	Arginine vasopressin
BAP	biological antioxidant potential
BDNF	Brain-derived neurotrophic factor
BH_4_	Tetrahydrobiopterin
BNP	B-type natiuretic peptide
CCL2	CC-chemokine ligand 2
cGMP	Cyclic GMP
CNP	C-type natiuretic peptide
CNS	Central nervous system
CO	carbon monoxide
CRH	Corticotrophin-releasing hormone
CXCL1	CXC-chemokine ligand 1
d-ROMs	derivatives of reactive oxygen metabolites
EAE	experimental autoimmune encephalomyelitis
ER	estrogen receptor
ETC	electron transport chain
FSH	Follicle Stimulating Hormone
GABA	gamma-aminobutyric acid
GAD	glutamic acid decarboxylase
GC	guanylyl cyclase
Gpxs	glutathione peroxidases
GR	Glucocorticoid receptor
HO-1	Heme oxygenase 1
HPA	Hypothalamic-pituitary-adrenal
HSD	hydroxysteroid dehydrogenase
IDO	indoleamine 2,3-dioxygenase
IFN-γ	interferon-gamma
IL	Interleukin
IL-1RA	interleukin-1 receptor antagonist
IL-1β	Interleukin-1beta
InhA	inhibin A
InhB	inhibin B
Keap1	Kelch-like ECH-associated protein 1
LPS	lipopolysaccharide
LTP	long-term potentiation
MAO-A	Monoamine oxidase-A
MAOIs	Monoamine oxidase inhibitors
MAPK	mitogen-activated protein kinase
MDA	Malonaldehyde
MDD	Major Depressive Disorder
MR	Mineralocorticoid receptor
mTORC1	Mammalian target of rapamycin complex-1
NE	norepinephrine
NMDA	N-methyl-D-aspartate
NO	nitric oxide
Nps	natiuretic peptides
Nrf2	nuclear respiratory factor 2
O^2−^	oxide ion
ox LDL	oxidized lipoproteins
PFC	Prefrontal cortex
PLCγ1	phospholipase Cγ1
POMC	Pro-opiomelanocortin mRNA
PVN	Paraventricular nucleus
RAAS	renin–angiotensin–aldosterone system
RNS	reactive nitrogen species
ROS	reactive oxygen species
SAR	superoxide anion radicals
SERM	Selective estrogen receptor modulators
sMaf	small musculoaponeurotic fibrosarcoma
SNRIs	Serotonin/norepinephrine reuptake inhibitors
SSRIs	Selective serotonin reuptake inhibitors
TAK1	TGF-β activated kinase 1
TCAs	Tricyclic antidepressants
TNF-α	Tumor necrosis factor-alpha
TrkB	Tyrosine kinase receptor B
